# Is loneliness a bridge to digital escape? A path analysis on psychological symptoms and internet addiction

**DOI:** 10.3389/fpsyg.2026.1816905

**Published:** 2026-05-01

**Authors:** Okan Bilgin, Murat İnce, Özgür Murat Çolakoğlu

**Affiliations:** 1 Ereğli Faculty of Education, Zonguldak Bülent Ecevit University, Zonguldak, Türkiye; 2 Faculty of Education, Yildiz Technical University, İstanbul, Türkiye

**Keywords:** internet addiction, loneliness, psychological symptoms, structural equation modeling, university students

## Abstract

**Introduction:**

Internet addiction has become a significant public health concern that threatens individuals’ psychological well-being. This study aimed to examine the effects of psychological symptoms (somatization, hostility, negative self-concept, depression, and anxiety) on internet addiction and to explore the mediating role of loneliness.

**Methods:**

Using a correlational survey design, data were collected from 1,404 university students across different academic disciplines. Psychological symptoms, internet addiction, and loneliness were measured using the Brief Symptom Inventory, the Internet Addiction Scale, and the ULS-8 Loneliness Scale. Structural equation modeling with bootstrapping procedures was employed.

**Results:**

The results indicated that participants reported moderate levels of internet addiction and low levels of loneliness. The effects of somatization, hostility, negative self-concept, and depression on internet addiction were fully mediated by loneliness. Negative self-concept showed the strongest effect on loneliness. In contrast, anxiety did not have a significant effect on loneliness but exerted a strong direct effect on internet addiction.

**Discussion:**

These findings suggest that loneliness may function as a key intermediary variable linking psychological symptoms to internet addiction, whereas anxiety may exert a more direct influence. The results highlight the importance of tailored interventions addressing both loneliness and anxiety in preventing internet addiction.

## 1 Introduction

In the twenty-first century, internet technologies have become an integral part of everyday life, shaping activities ranging from information access to social interaction. However, the widespread and pervasive use of the internet has also brought increasing attention to concepts such as problematic internet use (PIU) and internet addiction, which are characterized by a loss of control over online activities and are associated with impairments in academic, social, and psychological functioning (Fang et al., 2023; Moretta and Buodo, 2020). With the global number of internet users exceeding four billion, university students and adolescents represent particularly vulnerable groups due to the developmental sensitivity of identity formation and socialization processes during these life stages (Cai et al., 2023). A growing body of research has consistently demonstrated strong associations between problematic internet use and a range of psychopathological symptoms, including depression, anxiety, loneliness, and reduced subjective well-being (Bousono et al., 2017; Haddad et al., 2021; Xie et al., 2025). Despite this well-established link, the mechanisms through which psychological factors are associated with internet addiction—and the complex interplay among these factors—remain insufficiently clarified, particularly with regard to which symptoms have direct effects on internet addiction, and which operate indirectly through intermediary pathways.

One of the most influential models explaining the etiology of internet addiction is Davis’s (2001) cognitive–behavioral model, which conceptualizes psychosocial difficulties such as depression, anxiety, and loneliness as distal yet necessary antecedents of problematic internet use. According to this model, individuals may turn to the internet as a coping mechanism to escape real-life stressors, regulate negative affect, or compensate for unmet social needs. From the perspective of the self-medication hypothesis, individuals experiencing psychological distress seek temporary relief through the immediate rewards and perceived anonymity provided by online environments.

The existing literature consistently indicates that internalizing problems, particularly depression and negative self-concept, are positively associated with internet addiction (Dalbudak and Evren, 2014; Richman et al., 2016). Individuals with such vulnerabilities are more likely to prefer online interactions over face-to-face communication, where they may perceive greater control and reduced risk of rejection (Kim et al., 2009). In particular, individuals with a negative self-concept often view the online environment as a safer interpersonal space due to heightened fears of social rejection, which in turn increases the risk of developing maladaptive patterns of internet use (Moretta and Buodo, 2020). However, whether all psychological symptoms operate through the same underlying mechanisms in the pathway to internet addiction remains an open and clinically relevant question, with important implications for the design of targeted intervention strategies.

Loneliness has emerged as a key mediating construct in the process through which psychological symptoms are associated with internet addiction. According to the social compensation hypothesis, individuals with limited social skills, depressive tendencies, or low self-esteem may use the internet as a compensatory tool to address deficiencies in their offline social relationships (Kim et al., 2009; Xie et al., 2025). Importantly, loneliness does not merely reflect the quantitative absence of social ties but represents a subjective and distressing experience arising from a perceived discrepancy between desired and actual social relationships (Kahraman, 2018; Cornwell and Waite, 2009).

A growing body of empirical research indicates that loneliness plays a full or partial mediating role in the association between depression and internet addiction (Xie et al., 2025; Wongpakaran et al., 2021; Moretta and Buodo, 2020). For instance, individuals exhibiting depressive symptoms or a negative self-concept often experience social withdrawal and heightened feelings of loneliness, which subsequently motivates increased internet use as a means of alleviating loneliness, seeking social connection, or providing distraction from negative affect (van Winkel et al., 2017). In this sense, loneliness functions as a psychological bridge that channels underlying vulnerabilities into maladaptive patterns of internet use. However, contrary to this prevailing assumption, it remains unclear whether affective states characterized by high levels of physiological and emotional arousal—such as anxiety—follow the same loneliness-mediated pathway or operate through a distinct mechanism in the development of internet addiction.

Although the association between anxiety and internet addiction is well established (Cho and Shin, 2013; Azher et al., 2014), the nature of this relationship may differ from that observed for depression. Previous research suggests that anxiety is often linked to impulse control difficulties and obsessive thought patterns, independent of loneliness, and that internet use may serve not as a means of social connection but rather as a direct tool for tension reduction and avoidance (Ko et al., 2006; Weinstein and Lejoyeux, 2010; Tao and Liu, 2009). According to Caplan’s (2010) model, problematic internet use is closely related to deficits in emotion regulation. Individuals with elevated anxiety levels may therefore engage in compulsive internet use—such as gaming or prolonged browsing—to immediately alleviate intense anxiety, somatic complaints, and physiological arousal, even in the absence of perceived loneliness (Cai et al., 2023).

Preliminary analyses of the present study, together with emerging evidence from the literature, suggest that anxiety may be directly associated with internet addiction, rather than being mediated by loneliness. This pattern suggests that anxiety-related internet addiction may be primarily related to difficulties in emotion regulation rather than social deprivation or perceived social isolation. In contrast, symptoms such as depression, hostility, and negative self-concept tend to precipitate social withdrawal and loneliness, which subsequently foster maladaptive internet use (van Winkel et al., 2017; Cacioppo et al., 2014; Qualter et al., 2013). Clarifying this distinction is of critical importance for the development of individualized and mechanism-based clinical interventions.

Recent empirical studies further support the notion that anxiety-related digital behaviors may follow distinct pathways compared to other psychological symptoms. For example, Li et al. (2025) demonstrated a bidirectional longitudinal relationship between anxiety and problematic short-form video use, highlighting the role of impulsivity and reduced delay of gratification. Similarly, Yang et al. (2025), using cross-lagged panel network analysis, reported that anxiety-related symptoms may function as direct antecedents of mobile phone addiction rather than being fully mediated by social factors. In addition, Todorovic et al. (2025) identified bidirectional associations between problematic digital use and mental health difficulties, suggesting complex and dynamic interactions that extend beyond simple mediation frameworks. From a social perspective, Zhou et al. (2024) emphasized that disruptions in social relationships are strongly associated with internet addiction, thereby supporting the role of loneliness as a relevant intermediary variable for certain psychological profiles. Moreover, Lee and Won (2025) highlighted the importance of social and contextual factors in problematic digital behaviors, further underscoring that different psychological mechanisms may underlie distinct patterns of internet use. Taken together, these findings provide contemporary empirical support for the view that anxiety may be linked to internet addiction through more direct and regulation-based processes, whereas other psychological symptoms may operate through socially mediated pathways such as loneliness.

Many previous studies in this field have relied on relatively small samples or simple regression-based analytical approaches. In contrast, the present study draws on a large sample of 1,404 university students and employs structural equation modeling with bootstrapping procedures, allowing for the simultaneous examination of complex networks of direct and indirect associations while minimizing measurement error. This analytical strategy enables a more robust and reliable assessment of the strength and direction of relationships among psychological symptoms, loneliness, and internet addiction.

Beyond addressing the general question of what contributes to internet addiction, the present study seeks to answer a more specific and clinically meaningful question: Which psychological symptoms are associated with internet addiction, and through which pathways do these associations operate? The findings are expected to provide evidence-based guidance for preventive interventions by highlighting the need for social integration and loneliness-reduction strategies for individuals with depressive symptoms and low self-esteem, as well as direct emotion regulation and anxiety management strategies for individuals with elevated anxiety.

Accordingly, the main objectives of this study are to determine the levels of loneliness, internet addiction, and psychological symptoms among university students; to examine the relationships among these variables; and to explore the potential mediating role of loneliness in the association between psychological symptoms and internet addiction. In line with these objectives, the following research questions were addressed:

1 What are the levels of loneliness, internet addiction, and psychological symptoms among university students?
2 Are there significant relationships among loneliness, internet addiction, and psychological symptoms?
3 Does loneliness play a potential mediating role in the association between psychological symptoms and internet addiction?

## 2 Materials and methods

### 2.1 Participants and procedure

This study was designed using a cross-sectional correlational research model aimed at examining the structural relationships among the study variables. To examine the potential mediating role of loneliness in the relationship between psychological symptoms (independent variables) and internet addiction (dependent variable), path analysis was employed, allowing for the simultaneous examination of effects among observed variables. The complex pattern of relationships among the variables was tested using structural equation modeling (SEM).

The study population consisted of university students. Participants were recruited using a convenience sampling method, which allowed for efficient data collection in terms of time and accessibility. The study sample comprised a total of 1,404 university students enrolled in different academic departments and grade levels. With regard to demographic characteristics, 70.8% of the participants were female (*n* = 994) and 29.2% were male (*n* = 410).

Data were collected from university students enrolled in various faculties using a convenience sampling method through face-to-face administration in classroom settings. The data collection process was conducted between 16 September 2024 and 24 March 2025. In line with the ethical standards of the Declaration of Helsinki, participants were informed about the purpose of the study, and written informed consent was obtained on a voluntary basis prior to participation. Following data collection, preliminary screening indicated that there were no missing data in the dataset; therefore, the entire dataset was included in the analyses.

This study was conducted in accordance with the ethical principles outlined in the Declaration of Helsinki (1964) and its subsequent revisions. Ethical approval was obtained from the Human Research Ethics Committee of Zonguldak Bülent Ecevit University (Protocol No: 231; Approval Date: 03 June 2024). Participation in the study was entirely voluntary, and informed consent was obtained from all participants prior to data collection. Participants were clearly informed that their responses would be treated with strict confidentiality and that their personal information would be protected throughout the research process. No procedures were implemented that could pose physical or psychological risk to the participants, and all stages of data collection were carried out with careful attention to ethical responsibility.

### 2.2 Materials

#### 2.2.1 Brief Symptom Inventory (BSI)

Psychological symptom levels were assessed using the Brief Symptom Inventory (BSI), originally developed by Derogatis (1992) and adapted into Turkish by Sahin and Durak (1994). The instrument consists of 53 items rated on a 5-point Likert scale ranging from 0 (not at all) to 4 (extremely). In line with the recommendations of Şahin and Durak, the Turkish version was administered using a five-factor structure rather than the original nine-factor solution. Accordingly, the scale comprises five subdimensions: Anxiety (13 items), Depression (12 items), Negative Self-Concept (12 items), Somatization (9 items), and Hostility (7 items). In the present study, internal consistency coefficients (Cronbach’s alpha) for the subscales were 0.86 for Anxiety, 0.87 for Depression, 0.87 for Negative Self-Concept, 0.80 for Somatization, and 0.78 for Hostility.

#### 2.2.2 Internet Addiction Scale

Internet addiction was measured using a 9-item, single-factor scale developed based on the DSM-5 diagnostic criteria for Internet Gaming Disorder developed by Taş and Bilgin (2018). In the scale development study, the single-factor structure was reported to explain 39.9% of the total variance, with factor loadings ranging from 0.548 to 0.707. In the present sample, the internal consistency coefficient (Cronbach’s alpha) of the scale was 0.80.

#### 2.2.3 ULS-8 Loneliness Scale

Loneliness was assessed using the short form of the UCLA Loneliness Scale (ULS-8), originally developed by Russell et al. (1980) and adapted into Turkish by Doğan et al. (2011). The scale consists of 8 items rated on a 4-point Likert scale ranging from 1 (never) to 4 (always) and yields a single-factor structure. In the present study, Cronbach’s alpha reliability coefficient of the ULS-8 was 0.72.

### 2.3 Data analysis

Data analyses were conducted using SPSS version 22.0 and AMOS version 21.0. Prior to main analyses, the dataset was examined for missing values and outliers. Multivariate outliers were assessed using Mahalanobis distance values. To evaluate the assumption of normality, skewness and kurtosis values were inspected. The skewness values for somatization, anxiety, depression, hostility, negative self-concept, and loneliness were positive, and the data did not meet the assumption of multivariate normality, as indicated by Mardia’s multivariate kurtosis (35.037; c.r. = 20.992).

Due to the violation of the multivariate normality assumption, parameter estimates were obtained using the bootstrap maximum likelihood (Bootstrap ML) method. In this procedure, 1,000 bootstrap samples were generated through resampling from the original dataset, and confidence intervals for parameter estimates were calculated based on the resulting bootstrap distributions. Model fit was evaluated using multiple goodness-of-fit indices, including the Chi-square/df ratio (CMIN/df), GFI, AGFI, NFI, TLI, CFI, RMSEA, and SRMR. The interpretation of relationships among variables was guided by Cohen’s effect size criteria (Cohen, 1992).

For the path analysis, model fit was examined using the Bootstrap ML approach. The initially hypothesized model was labeled Model A, whereas the revised model obtained after model modifications was labeled Model B. The results of the structural equation modeling analyses were structured around the comparison of these two models. Statistical significance was evaluated at the 0.05 level.

## 3 Results

This section presents the findings obtained in line with the primary objectives of the study. In this context, descriptive statistics addressing the first research question—“What are the levels of internet addiction, loneliness, and psychological symptoms among university students?”—are presented in Table 1.

**Table 1:** Descriptive statistics for loneliness, internet addiction, and psychological symptoms.

Variable	*N*	Min	Max	*M*	SD
Internet addiction	1,404	1.00	5.00	2.30	0.63
Loneliness		1.00	4.00	1.58	0.47
Somatization		0.00	3.89	0.88	0.65
Hostility		0.00	4.00	1.25	0.74
Negative self-concept		0.00	3.67	0.95	0.71
Depression		0.00	3.92	1.33	0.77
Anxiety		0.00	3.46	0.94	0.65

*N* = 1,404; Min, minimum; Max, maximum; *M*, mean; SD, standard deviation.

As shown in Table 1, university students reported moderate levels of internet addiction (*M* = 2.30, SD = 0.63) and low levels of perceived loneliness (*M* = 1.58, SD = 0.47). Among the psychological symptoms, depression yielded the highest mean score (*M* = 1.33), whereas somatization showed the lowest mean level (*M* = 0.88). The correlation coefficients among internet addiction, loneliness, and psychological symptoms are presented in Table 2.

**Table 2:** Pearson correlations among study variables.

Variable	1	2	3	4	5	6	7
Somatization (1)	—						
Hostility (2)	0.59*	—					
Negative self-concept (3)	0.64*	0.68*	—				
Depression (4)	0.66*	0.67*	0.80*	—			
Anxiety (5)	0.72*	0.72*	0.82*	0.81*	—		
Internet addiction (6)	0.32*	0.36*	0.40*	0.38*	0.42*	—	
Loneliness (7)	0.26*	0.27*	0.48*	0.41*	0.39*	0.23*	—

^*^Values represent Pearson correlation coefficients; *p* < 0.05.

When the relationships among the variables were examined (see Table 2), all psychological symptoms were found to be positively and statistically significantly associated with both internet addiction and loneliness (*p* < 0.05). Anxiety showed the strongest correlation with internet addiction (*r* = 0.42), whereas negative self-concept demonstrated the strongest association with loneliness (*r* = 0.48). These preliminary findings are consistent with the hypothesized structural model, suggesting that anxiety may be more directly associated with internet addiction, whereas negative self-concept may be more strongly associated with internet addiction through loneliness.

### 3.1 Structural equation modeling and model fit assessment

**Figure 1:**
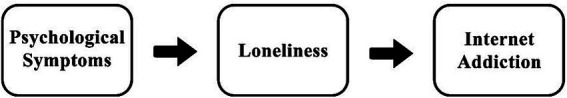
The initially proposed structural model (model A).

To examine the mediating role of loneliness in the effects of psychological symptoms on internet addiction, the initially hypothesized model (Model A), presented in Figure 1, and the final model obtained after model modifications (Model B) were tested using the bootstrap maximum likelihood method.

The analysis indicated that the goodness-of-fit indices for Model A were below acceptable thresholds (*χ*^2^/df = 44.86, CFI = 0.96, RMSEA = 0.18). Examination of the modification indices suggested that anxiety was more appropriately modeled as having a direct effect on internet addiction rather than an indirect effect through loneliness, as the path coefficient between anxiety and loneliness was not statistically significant (*p* > 0.05).

Accordingly, Model B was tested by adding the direct path from anxiety to internet addiction and removing nonsignificant paths. As presented in Table 3, Model B demonstrated excellent model fit (*χ*^2^/df = 4.41, CFI = 0.99, GFI = 0.99, RMSEA = 0.05, SRMR = 0.02). These results are consistent with the proposed theoretical model. However, it is important to note that Model B was derived through post-hoc adjustments based on modification indices and the removal of non-significant paths. Therefore, the findings should be interpreted as exploratory and hypothesis-generating rather than strictly confirmatory.

**Table 3:** Goodness-of-fit indices for model A and model B.

Fit index	Acceptable fit	Excellent fit	Model A	Model B
Chi-square/df (CMIN/df)	≤5	≤2	44.87	4.41
Goodness of fit index (GFI)	≥0.90	≥0.95	0.96	0.99
Adjusted goodness of fit index (AGFI)	≥0.90	≥0.95	0.77	0.98
Normed fit index (NFI)	≥0.90	≥0.95	0.96	0.99
Tucker-Lewis index (TLI)	≥0.90	≥0.95	0.85	0.99
Comparative fit index (CFI)	≥0.90	≥0.95	0.96	0.99
Root mean square residual (SRMR)	≤0.08	≤0.05	0.12	0.02
Root mean square error of approximation (RMSEA)	≤0.08	≤0.05	0.18	0.05

Values are based on bootstrap maximum likelihood estimation.

### 3.2 Direct and indirect effects (path analysis results)

An examination of the standardized path coefficients (*β*) and significance levels obtained from the final model (Model B) revealed distinct underlying mechanisms linking psychological symptoms to internet addiction.

### 3.3 Indirect effects via loneliness

The effects of somatization, hostility, negative self-concept, and depression on internet addiction were found to be fully mediated by loneliness. These variables did not have significant direct effects on internet addiction; instead, their effects operated indirectly through loneliness. Notably, negative self-concept exhibited the strongest effect on loneliness (*β* = 0.50), suggesting that impairments in self-perception may be linked to increased feelings of loneliness. In turn, loneliness had a positive and significant effect on internet addiction (*β* = 0.08).

### 3.4 Direct effect (the distinct role of anxiety)

In contrast to the other psychological symptoms, anxiety did not have a significant effect on loneliness. Rather, anxiety had a strong and direct effect on internet addiction (*β* = 0.39). This finding suggests that individuals with elevated anxiety levels may engage in internet use as a direct coping or avoidance strategy, even in the absence of perceived loneliness, indicating that anxiety may follow a pathway distinct from other psychological symptoms.

**Figure 2:**
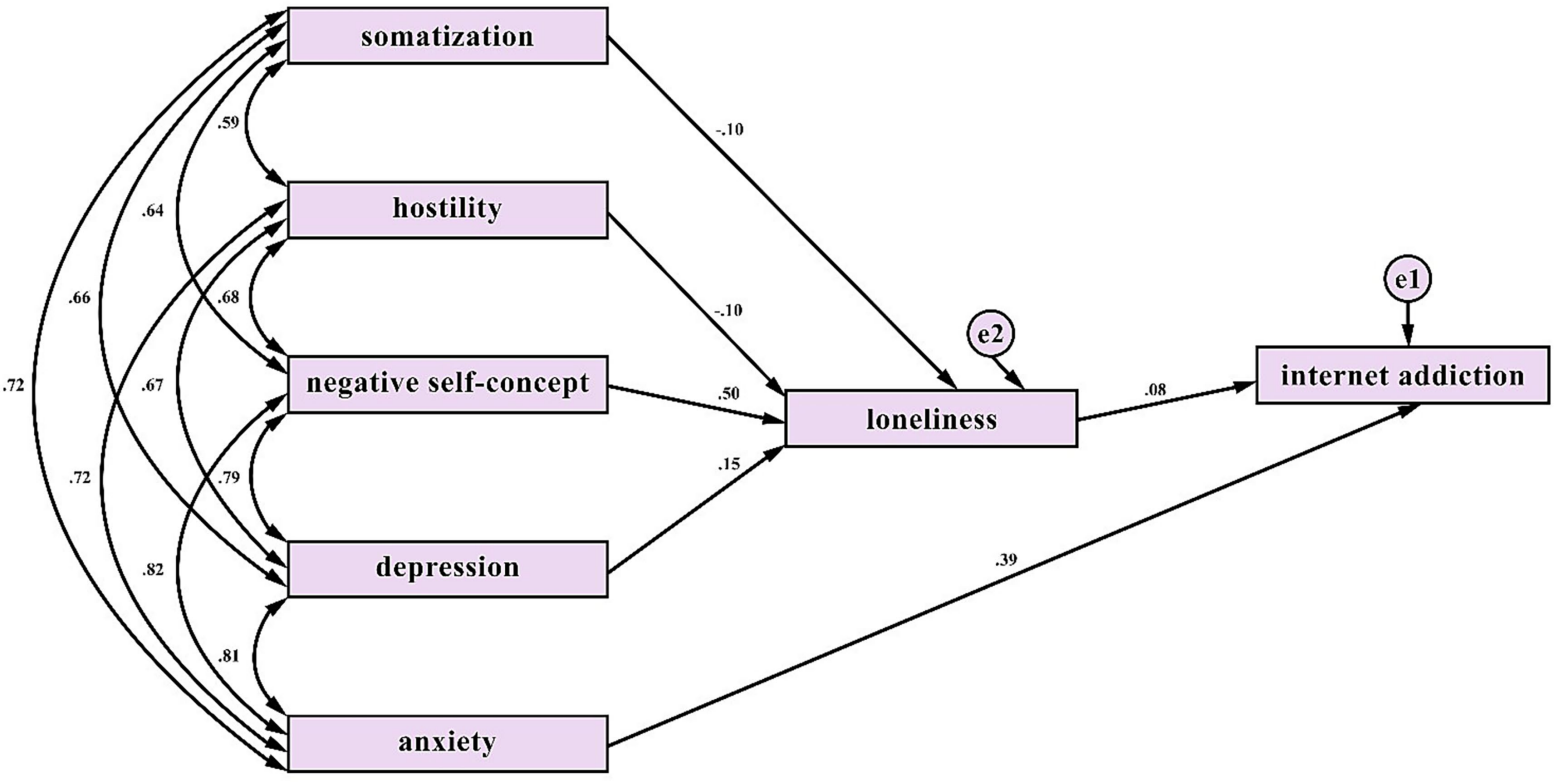
The final structural path model after model modifications (model B).

Bootstrap analyses further indicated that the confidence intervals for both direct and indirect effects did not include zero, suggesting that all parameter estimates were statistically significant at the 95% confidence level. Overall, these findings suggest that while somatization, hostility, depression, and negative self-concept influence internet addiction through a loneliness-based pathway, anxiety exerts a direct effect on internet addiction, independently of loneliness. The final modified model is illustrated in Figure 2.

The findings regarding the total, direct, and indirect effects obtained from the final model are presented in Table 4.

**Table 4:** Total, direct, and indirect effects of psychological symptoms on internet addiction.

Independent variable	Mediator	Dependent variable	Total effect	Direct effect	Indirect effect
Somatization	Loneliness	Internet addiction	0.01	0.00	0.01
Hostility			0.01	0.00	0.01
Anxiety			0.39	0.39	0.00
Negative self-concept			0.04	0.00	0.04
Depression			0.01	0.00	0.01

The terms “effect” refer to standardized path coefficients derived from structural equation modeling. Due to the cross-sectional design, these statistical effects should be interpreted as patterns of association consistent with the hypothesized model rather than definitive evidence of temporal causation.

These findings suggest that loneliness may function as an intermediary variable linking various psychological symptoms to internet addiction, whereas certain symptoms, such as anxiety, may exert a direct effect on internet addiction.

## 4 Discussion

The present study examined the associations between psychological symptoms—namely somatization, hostility, negative self-concept, depression, and anxiety—and internet addiction (IA) among university students, with a particular focus on the potential mediating role of loneliness. The findings indicate that psychopathological factors involved in the etiology of IA do not appear to operate through a single mechanism. Rather, two distinct pathways emerged: a loneliness-oriented pathway grounded in social compensation processes and a direct emotion regulation–oriented pathway primarily associated with anxiety.

The most striking finding of the study is that anxiety was strongly and directly associated with internet addiction (*β* = 0.39), rather than indirectly through loneliness. Although previous research has consistently documented an association between anxiety and IA (Gu et al., 2024; Santos et al., 2017), this relationship has often been conceptualized as operating through mechanisms similar to those observed for depression, such as social withdrawal and isolation. In contrast, the present findings suggest that anxiety contributes to IA through an impulsive avoidance mechanism, independent of perceived loneliness.

As noted by Gu et al. (2024), the association between anxiety and internet addiction may stem from the use of the internet as a safety behavior aimed at reducing momentary tension and distress. Unlike individuals with depressive symptoms, highly anxious individuals may not seek online social connection but instead engage in compulsive internet use—such as gaming or continuous information consumption—to escape obsessive thoughts, regulate physiological arousal, or regain a sense of control. Supporting this interpretation, Santos et al. (2017) reported in a clinical sample that reductions in anxiety were associated with decreases in IA symptoms. This pattern suggests that anxiety-related IA may be more closely aligned with the impulsive coping component of Brand et al.’s (2019) I-PACE model rather than with loneliness-based social compensation processes.

Another key finding of the study is that the associations between somatization, hostility, negative self-concept, and depression, and internet addiction were mediated by loneliness. This result provides strong support for the Social Compensation Hypothesis of Internet Use. Consistent with this interpretation, Zhou et al. (2025), in their large-scale study involving over 131,000 participants, emphasized loneliness as the most critical mediating variable transforming psychological vulnerability into internet addiction.

In particular, the strong association between negative self-concept and loneliness (*β* = 0.50) aligns with Richman et al.’s (2016) unclear self-theory. Individuals with negative self-schemas are more likely to experience fear of rejection in offline interactions, leading to social inhibition and loneliness. To alleviate these feelings and present an idealized version of the self, such individuals may turn to online environments. Similarly, as noted by Kahraman (2018), loneliness experienced within narcissistic or avoidant personality patterns may push individuals toward the more controllable and less threatening relationships offered by virtual contexts. Recent findings by Xie et al. (2025) further corroborate the central mediating role of loneliness in the relationship between depression and IA, while highlighting the protective function of social support.

The indirect association between somatization and internet addiction via loneliness may be related to the tendency of individuals with physical complaints to experience reduced mobility or heightened health anxiety, which may contribute to social isolation and increased reliance on online activities. Likewise, individuals with high levels of hostility may encounter frequent interpersonal conflicts in face-to-face interactions, leading to diminished social support and loneliness. These individuals may subsequently gravitate toward online environments where aggressive impulses can be expressed or suppressed more anonymously (Vos et al., 2023; Nazzal et al., 2021; Koç, 2011).

The findings of the present study underscore the importance of moving beyond one-size-fits-all interventions in efforts to prevent and reduce internet addiction. Instead, intervention strategies should be symptom-specific and mechanism-based. It should also be noted that the indirect effects observed in this study were relatively small in magnitude. Although statistically significant due to the large sample size, their practical significance should be interpreted with caution. This suggests that loneliness may represent only one of several pathways linking psychological symptoms to internet addiction.

For individuals with elevated anxiety levels and heightened risk of IA, interventions should prioritize direct emotion regulation and anxiety management rather than merely restricting internet use. Cognitive Behavioral Therapy (CBT) programs focusing on emotion regulation skills, relaxation techniques, and impulse control may be particularly effective for this group. In such cases, social skills training may be less relevant than strategies aimed at managing anxiety-driven avoidance behaviors.

In contrast, for individuals exhibiting depressive symptoms, negative self-concept, or social withdrawal, the primary focus of intervention should be social integration. As suggested by Zhou et al. (2025), group-based activities, peer support programs, and social skills training that enhance social inclusion may reduce loneliness and, in turn, indirectly lower the risk of internet addiction.

Several limitations should be acknowledged. First, the study employed a cross-sectional design, which limits causal inferences regarding the directionality of the observed relationships. Therefore, the findings should be interpreted as reflecting associations rather than causal effects. In addition, alternative model configurations (e.g., internet addiction predicting loneliness or bidirectional relationships) are theoretically plausible. Future research employing longitudinal designs is needed to clarify the temporal ordering and potential bidirectional relationships among psychological symptoms, loneliness, and internet addiction (e.g., Pham et al., 2026; Dodan and Negru-Subtirica, 2025).

Second, the reliance on self-report measures may introduce response biases. Third, the sample consisted exclusively of university students, which may limit the generalizability of the findings. Finally, the internal consistency of the loneliness measure (ULS-8) was acceptable but relatively modest (*α* = 0.72), which should be considered when interpreting the findings.

Future studies should also differentiate between types of anxiety (e.g., social anxiety versus generalized anxiety) to further clarify their distinct roles in the development of internet addiction. Experimental and intervention-based research designs would additionally strengthen the evidence base for mechanism-specific prevention strategies.

This study offers empirical insights into the differentiation of mechanisms underlying the associations between psychopathological symptoms and internet addiction among university students. Specifically, anxiety was found to exert a direct effect on internet addiction, potentially reflecting emotion regulation and avoidance-related processes, whereas depression, negative self-concept, and somatization were exerted indirect effects on internet addiction through loneliness. By moving beyond the mere documentation of associations, these findings provide a structural perspective on how different psychological symptoms are associated with internet addiction, thereby contributing to a more nuanced and intervention-relevant understanding of this growing public health concern.

## Data Availability

The raw data supporting the conclusions of this article will be made available by the authors, without undue reservation.
